# Appraising the lifetime private economic returns of postgraduate degrees: Evidence from Pakistan

**DOI:** 10.1057/s41599-023-01565-6

**Published:** 2023-03-08

**Authors:** Adnan Bashir, Zahid Siddique

**Affiliations:** School of Social Sciences and Humanities (S3H), National University of Sciences and Technology (NUST), Islamabad, Pakistan

**Keywords:** Economics, Education, Development studies

## Abstract

In Pakistan, public sector investment has significantly increased in higher education over the last two decades. This study, therefore, aims to estimate the private economic returns of Ph.D. faculty members over their non-PhD counterparts in Pakistani universities. We use questionnaire-based survey data of 784 respondents comprising Ph.D. and non-Ph.D. faculty members. In the first step, earning function is estimated for the entire group. In the next step, the lifetime private economic returns are calculated with the help of the simulation process, which yields the net present values (NPV) of the lifetime earnings for the two subgroups. Our findings show that the lifetime private economic returns of a Ph.D. degree are higher than a non-Ph.D. degree. In the case of a domestic Ph.D. degree, the average lifetime economic returns of the Ph.D. are 46.5% higher than those of non-Ph.D. faculty members. In contrast, foreign-country Ph.D. degree holders earn 29.8% extra than non-Ph.D. faculty members. Therefore, the net lifetime returns of foreign Ph.D. holders are 16.7% points less than the domestic degree holders because the cost of doing a Ph.D. degree from a foreign university is higher. Our sensitivity analysis reveals that changing the retirement age from 60 to 55 and 65 does not affect these results. However, the difference between the net returns decreases if the retirement age is 55 and increases in the case of 65. Similarly, increasing the completion time of a Ph.D. also affects the net lifetime private economic return negatively.

## Introduction and background

The research on education as a form of investment and investigation of the role of human capital started in the 1960s. The interest in this area was enhanced by the observation that investment in human capital positively affects output, especially the rise in earnings per worker (Schultz 1961). The theory of human capital explains the impact of investment in human capital on an individual’s earning potential. Backer enriched this concept in 1964 by saying that an individual chooses that level of education where the present value of expected marginal returns of wages equals the marginal returns of other investments (Becker, 1964). Education is treated as an essential component of the human capital theory, where individuals are the investors in education, not the government. As a result, individuals recognize that further education will contribute to annual earnings. However, there is a tradeoff between education and working lifespan. Hence, the economic returns of educational investment inspire individuals to make deliberate and thoughtful decisions regarding further investment in education.

**Fig. 1:**
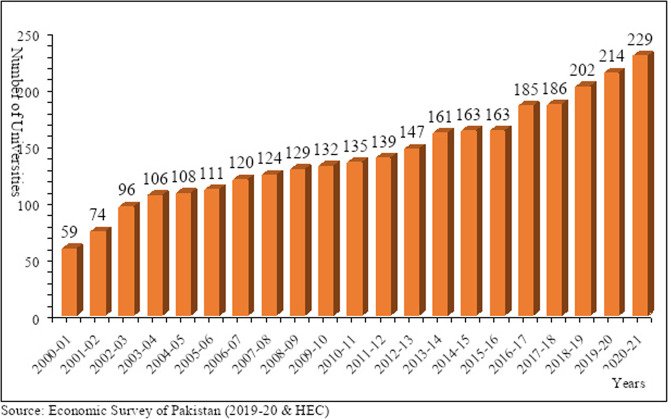
Number of recognized universities in Pakistan. The total number of registered universities in the Higher Education Commission (HEC) of Pakistan till 2021.

The literature on returns to higher education indicates that schooling positively impacts an individual’s lifetime earnings (Poteliene and Tamasauskiene, 2015). Studies have focused on the private economic returns for various levels of education, especially higher education (Hines et al., 1970; Mincer, 1974; Romanello, 2017; Nasir, 1998; Okuwa, 2004; Bashir and Iqbal, 2010; Patrinos et al., 2021; Fatima and Tahira, 2014). This provides support to the idea that investment in education would positively contribute to private economic returns, implying that higher education is an essential mainstay of every nation to improve their living standards. Looking at the evolution of the higher education sector in Pakistan, in the initial 54 years from 1947 to 2001, the country had only 59 universities. Pakistan’s Higher Education Commission (HEC) was established in 2001-02 as an autonomous organization independent of the Federal Ministry of Education. As a result of its efforts, the total number of public and private universities increased to 50 and 37 within six years of establishment, respectively (Hoodbhoy, 2009). The total number of universities in the previous decade increased by 78% (Mahmood, 2016). In 2021, the registered universities in the HEC had escalated to 229 (See Fig. 1), including 140 and 89 public and private universities, respectively (HEC, 2021).

Numerous studies have attempted to estimate the private economic returns at different levels of education in Pakistan (Haroon, 2015; Afzal, 2011). Along with the number of universities, the country has also seen significant growth in the teaching community, with the number of faculty members increasing from 5988 to 47,396 in just two decades (Table 1). Parveen et al. (2011) explored the various dimensions of reforms in Pakistan over time, such as the issues faced by Pakistan’s education system until the establishment of HEC (Table 2).

**Table 1:** Number of Ph.D. and non-Ph.D. Faculty Members in the Universities.

Provinces	Sectors	Female		Male		Total
		Non-Ph.D.	Ph.D.	Non-Ph.D.	Ph.D.	
Punjab	Public	3662	1251	3339	2961	11213
	Private	1865	323	2959	1075	6222
Federal	Public	1938	608	2869	2368	7783
	Private	736	59	795	315	1905
Sindh	Public	1672	636	2970	1320	6598
	Private	2120	256	2585	599	5560
KPK	Public	864	280	1805	1734	4683
	Private	276	39	907	407	1629
Baluchistan	Public	548	70	833	284	1735
	Private	12	2	27	27	68
**Total**		**13,693**	**3,524**	**19,089**	**11,090**	**47,396**

Source: Higher Education Commission of Pakistan (2017–18).

**Table 2:** Distribution of sampled respondents by disciplines.

Gender	Area of study				
	Social sciences	Mang. sciences	Engineering & computer sciences	Natural sciences	Total
Male	145	102	113	162	522
Female	77	56	50	79	262
**Total**	**222**	**158**	**163**	**241**	**784**

**Source:** Author’s Estimation from the survey data.

The private sector universities are 38% of the total number of universities, whereas the faculty members in the private sector universities are 33% of the total faculty members (HEC, 2017–18).

**Fig. 2:**
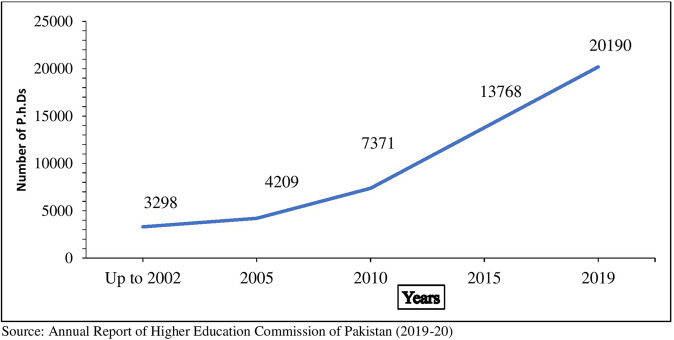
Number of Ph.D. produced by Pakistani Universities over time. The vertical and horizontal axis explains the number of Ph.D. degree holders and time frame, respectively. It shows an increasing trend in the number of Ph.D. degree holders over time.

The Pakistani universities produced 3,298 Ph.D. degree holders before the commencement of HEC. In contrast, as per the latest data, 20,190 people have Ph.D. degrees in Pakistan, (See Fig. 2), (HEC, 2019–20). This means that more than 72% (14,614/20,190) of Ph.D. degree holders (see Table 1 and Fig. 2) are working in the higher education sector as faculty members, and it is imperative to estimate the difference in lifetime economic returns at different levels of education. Many papers have compared returns to educational investment at different levels of education (Khan and Irfan, 1985; Blundell et al. 2000; Becker, 1975; Wahrenburg and Weldi, 2007; Qazi et al. 2010; Nazar and Chaudhry, 2017).

This study aims to determine the lifetime private economic returns of postgraduate degrees. The study also concentrates on the impact of educational investment on individual private returns of Ph.D. over non-Ph.D. faculty in Pakistani universities. Furthermore, it also investigates the private economic returns of Ph.D. over non-Ph.D. faculty and those with Ph.D. degrees from domestic and foreign country universities. Finally, the sensitivity analysis is carried out to determine the impact of relaxation in three assumptions on these private economic returns: (a) the impact of one extra year of completing the Ph.D. degree, (b) the impact of changes in retirement age and (c) the effects of varying interest rates.

The prior studies have examined the private rate of returns at different levels of education, including primary, secondary, matric, intermediate, etc. Moreover, previous studies focused on the impact of one extra year of schooling on private returns. This research calculates the lifetime private economic returns of Ph.D. over non-Ph.D. faculty members working in the higher education sector in Pakistan.

The study focuses on these objectives:

- To estimate the returns to educational investment at the higher education level in Pakistan to find the lifetime economic returns of Ph.D. over non-Ph.D. faculty members in the education sector of Pakistan.
- Compare the lifetime economic returns of Ph.D. faculty members who have Ph.D. degrees from domestic and foreign universities.

The rest of the paper is organized as follows. Section 2 presents the results of the studies that have already been conducted. Section 3 explains the data set, sample size, and the estimation technique used in our analysis. The preliminary results are analyzed in the first part of section 4. The second part has an empirical analysis that presents the difference in the rate of returns of Ph.D. over non-Ph.D. faculty members in Pakistan. Finally, the summary and discussion of the estimated results are exhibited in section 5.

## Literature review

This section covers a brief review of the prior literature on the private economic returns of educational investment in different levels of education. Becker (1964) considers educational expenditures as an investment in human capital and examines the rate of return at the higher education level. Therefore, the rate of return of each degree appeals to the candidate to get admission because the attractive remuneration will play a vital role in engaging in that degree. Otherwise, the candidate will enter the labor market. Becker, in 1974, further identifies that private returns are the primary motivators for getting engaged in further studies. Similarly, the theoretical and empirical earning model of Mincer (1974) presents the impact of investment in schooling on private earnings with a critical assumption that human capital investment becomes zero once a person enters the labor market after education.

Meanwhile, there is a tradeoff between schooling years and the working life span, so the lifetime earnings will decrease with one extra year of schooling. Yu et al. (2022) examine the impact of education on productivity. The control variables of the study are average years of schooling, primary and secondary years of schooling, and tertiary years of schooling. The study results suggest that priority must be given to human capital, which is not only essential for the education status of society but also helpful for productivity.

Psacharopoulos (1985) analyzes the returns to investment in education using the data of 60 countries, whereas the higher level of education and high skills are the main drivers for increasing private returns in education. Later on, Becker (1993) provided a clear picture of human capital investment because the focus of the study was to find the impact of human capital rather than machinery or physical capital. Meanwhile, physical capital is more important to maintain consistency in economic growth, but human capital plays a vital role in improving workers’ skills.

Blundell et al. (2000) examine the impact of higher education on private financial and non-financial returns. The results show that the raw returns of the undergraduate degree are around 21% for men and 39% for women. Finally, Zoran (2015) examines the importance of knowledge for economic and development improvement. The results indicate that the level of education and educational system contribute to the economy through skilled staffing. Furthermore, a positive correlation has been established between public expenditures on education and economic growth. For example, in developed countries, economic growth increased by more than 1% due to a 1% increase in education spending, which is 0.77% in European countries. Mitra (2019) investigates the marginal rate of education returns to different education levels. The study uses the internal rate of return and earning function methods to estimate the rates of return.

Ganyaupfu (2014) estimates the private returns of higher education using the human capital theory, and the data were collected through a non-random sampling technique. Moreover, the results indicate a positive relationship between higher levels of education and private returns. Nazar and Chaudhry (2017) appraise the private returns to education, including 850 male and female respondents between 15 to 65 years. The results are calculated using the Mincer earnings function, where the estimation technique is an ordinary least square method. Therefore, there is a direct relationship between job switching and private returns. Javed and Arshad (2013) examine the impact of education on private returns through ordinary least-square methods. The primary study data contained data from three universities, and the findings give insight into the direct relationship between the level of education and the university’s faculty earnings.

Iqbal and Bashir (2010) investigate the private return rate at different education levels, which shows that returns of Ph.D. degrees would be increased by increasing the retirement age. The rate of return at the higher education level is estimated using the internal rate of return (IRR). The study results were based on the four levels of education, including Professional Bachelor, Master, M.Phil, and Ph.D., which shows that the rate of return for Master and M.Phil is higher than for the Bachelor and Ph.D. level. The direct and opportunity costs are the critical reasons for the low rate of return of a Professional Bachelor’s degree. Afzal et al. (2015) examine the impact of different levels of education on the earnings of employees working in financial institutions. Public sector financial institutes are paying higher wages than the private sector. Their results show that public sector employees receive 15.4% returns to education, while the returns to education in the entire sample are 12%. The returns to education are calculated with the help of the earnings function. Khan (2021) investigates the returns of higher education in Pakistan, clearly indicating that higher education’s economic return is higher than all other levels of education. The empirical results were investigated through regression analysis using earning function.

## Theoretical model and research methods

**Fig. 3:**
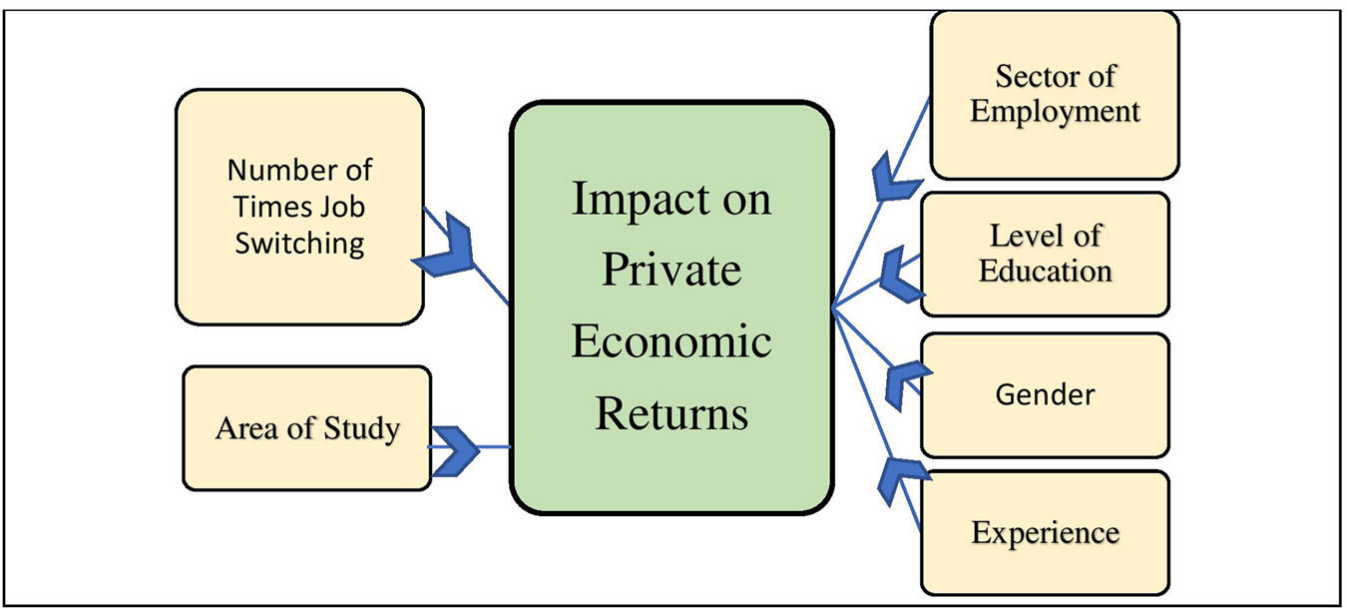
Conceptual framework of the study. It shows the effect of independent variables on private economic returns.

Education level determines the difference in private economic returns of individuals. Numerous factors impact the private economic returns of Ph.D. over non-Ph.D. faculty, including the study area, experience, level of education, degree country, gender, and the nature of work or job. As per the literature, the level of education and experience are the major factors contributing to the growth of private economic returns (See Fig. 3). But there is a tradeoff between experience and education because an extra year of schooling decreases a year from the working life span if the retirement age is fixed.

As per the literature, education costs are forgone earnings and direct costs, but investment in education pays higher returns. If these private economic returns are higher than foregone earnings and direct costs, investment in higher education is worthwhile. Suppose that education starts paying Y_j_ amount at time T* = t_j_ where j is the number of years of schooling.

For simplicity, it is assumed that education costs constantly increase at a certain level. Moreover, the cost of education will be different by the level of education with the J years of schooling. The study’s objective is to estimate the private economic returns of Ph.D. over non-Ph.D. faculty of Pakistan using the augmented human capital earning function. Initially, the earning function was based on years of schooling and professional experience, but the current study focuses on the level of education instead of the years of schooling that positively correlated with earnings. An individual’s lifetime earnings are based on the starting wages (Y_j_) and the annual wage increment (ΔY_j_).

The study estimates the rate of return of Ph.D. and non-Ph.D. faculty with 18 (J_1_) & 21 (J_2_) years of education. The starting wages of an individual with a Ph.D. (J2) and non-Ph.D. (J1) degrees are Y2 and Y1, and the constant annual increments are (∆*Y*2) & (∆*Y*1) till the retirement age (T), respectively. The initial point of Yj and (∆Yj) depends on the level of education. The lifetime earnings of an individual are given by the present value of expected lifetime earnings associated with education (*PV*_*E*_), given starting wage (Yj), and constant increment (∆*Yj*).

1 $$PV_{M.phil}^E = \left[ \begin{array}{l}Y_1\left( {\frac{1}{{\left( {1 + r} \right)^t}} + \frac{1}{{\left( {1 + r} \right)^{t + 1}}} + \cdots + \frac{1}{{\left( {1 + r} \right)^T}}} \right) + \\ \Delta Y_1\left( {\frac{1}{{\left( {1 + r} \right)^{t + 1}}} + \frac{2}{{\left( {1 + r} \right)^{t + 2}}} + \cdots + \frac{{T - t}}{{\left( {1 + r} \right)^T}}} \right)\end{array} \right]$$

2 $$PV_{Ph.D}^E = \left[ \begin{array}{l}Y_2\left( {\frac{1}{{\left( {1 + r} \right)^p}} + \frac{1}{{\left( {1 + r} \right)^{p + 1}}} + \cdots + \frac{1}{{\left( {1 + r} \right)^P}}} \right) + \\ \Delta Y_2\left( {\frac{1}{{\left( {1 + r} \right)^{p + 1}}} + \frac{2}{{\left( {1 + r} \right)^{p + 2}}} + \cdots + \frac{{P - p}}{{\left( {1 + r} \right)^P}}} \right)\end{array} \right]$$

There are no educational costs for all non-Ph.D. degree holders after “t” years of age, whereas the non-Ph.D. degree holders can join the labor market, but those students willing to pursue a Ph.D. will pay the extra cost for the next four years.

3 $$PV_{Ph.D}^C = \left[ {\left( \begin{array}{l}\frac{{C1}}{{\left( {1 + r} \right)^j}} + \frac{{\left( {C1 + \Delta C} \right)}}{{\left( {1 + r} \right)^{j + 1}}} + \frac{{\left( {C2 + 2\Delta C} \right)}}{{\left( {1 + r} \right)^{j + 2}}} + \\ \frac{{\left( {C3 + \left( {j - 1} \right)\Delta C} \right)}}{{\left( {1 + r} \right)^{j\prime }}}\end{array} \right)} \right]$$

## Empirical model

The study employs a modified version of earning function model of 1974,

4 $$\begin{array}{l}Y_i = \alpha + \beta _1NOW_i + \beta _2AOS_i + \beta _3MS_i + \beta _4FON_i + \beta _5Emp\,Sect_i \\\qquad\;+\; \beta _6S_i + \beta _7E_i + \beta _8E_i^2 + \beta _9Gender_i + \beta _{10}COD_i + \beta _{11}JS_i + u_i\end{array}$$

where Y_i_ is monthly earnings, Nature of work (NOW), Area of Study (AOS), Marital Status (MS), Funded vs. non-Funded (FON), Country of Degree (COD), Job Switch (JS), Employment Sector like a public or private, and Level of Study (S). The dependent variable of Eq. (4) is the average monthly income in current rupees after-tax, and E (Experience) is a change in income due to a one-year increase in working experience.

The level of education and working experience are negatively correlated because, holding the retirement age constant, those who study longer have a less working time span. Meanwhile, the level of education and working experience correlate positively with lifetime earnings, and the below-given method computes the years of experience,

5 $$E = A - S - B$$

A is the employee’s current age, S is the level of education, and B is starting age of education at grade 1 (i.e., six years). Our objective is to estimate the private rate of return of Ph.D. over non-Ph.D. faculty with the help of earning function. Moreover, the rate of return will be calculated by the NPV method, where the coefficient values of regression analysis help calculate the private return. In this study, “T” denotes retirement age (60 Years), while the starting working age for non-Ph.D. and Ph.D. faculty members are assumed to be 25 and 29 years, respectively. Therefore, the values of the coefficients are first estimated from Eq. (4). Then, those coefficient values are used to simulate the expected lifetime earnings of an individual. Finally, the discount rate will be applied to the undiscounted expected lifetime earnings to determine the NPV of expected lifetime earnings of a Ph.D. and non-Ph.D. faculty members.

## Data description

The study data were collected through a questionnaire survey, and the survey respondents were Ph.D. and non-Ph.D. faculty members of Pakistani universities. The survey respondents belonged to different cities in four provinces of Pakistan, including Islamabad, Rawalpindi, Lahore, Multan, Rahim Yar Khan, Bahawalpur, Faisalabad, Karachi, Jamshoro, Larkana, Quetta, Mardan, and Peshawar. The data were collected using a stratified random sampling technique where the total population is divided into subgroups known as strata, such as Ph.D. and non-Ph.D. faculty members, male and female, and public & private sector universities. The survey respondents were those faculty members who completed their terminal degrees from 2001 to 2020.

The survey respondents belong to the following five disciplines: social sciences, management sciences, engineering, computer sciences, and natural sciences. The sample size is 784 respondents from universities in different cities, containing 28.3%, 20.2%, 20.8%, and 30.7% share of the social sciences, management sciences, engineering & computer sciences, and natural sciences, respectively. The sample size computation was based on the following formula,

6 $$n = Z^2 \cdot \frac{{P\left( {1 - P} \right)}}{{e^2}}/1 + \left( {Z^{ \wedge 2} \cdot P\left( {1 - P} \right)/e^2 \cdot N} \right)$$

Where Z^^2^ is the critical value of the normal distribution at α/2 (e.g., for a confidence level of 95%, α is 0.05, and the critical value is 1.96), e^^2^ is the margin of error of 5%, P is the Sample proportion 0.5, and N is Population Size. The total population of the Ph.D. and non-Ph.D. faculty members in different Pakistani universities are 47,346, containing 14,614 Ph.D. and 32,782 non-Ph.D. faculty members (HEC, 2017–18). The computation of the sample size from the given population is selected by identifying the sample size separately for the Ph.D. and non-Ph.D. faculty members. The minimum number of required responses was 754 (380 & 374 responses from Ph.D. and non-Ph.D. faculty members). The data of male and female faculty members were collected as per the labor force participation rate mentioned in Pakistan Economic Survey, 2021-22 (Andlib and Khan, 2018).

## Definitions of variables

Numerous variables play a role in determining the rate of return for different levels of education. The definitions of selected variables are presented in Table 3.

**Table 3:** The definitions of the variables.

Variables	Definition of Variables
	**Dependent Variable**
Y	Monthly Earnings or Wages in Pakistani rupee (PKR)
	**Independent Variables**
Level of Education (Si)	A Dummy Variable: For Ph.D. = 1 and 0 otherwise
Experience (E)	Number of working years
Experience Square	Square of experience to capture the concavity of the age-earning profile
Job Switching (JS)	No Job Switched is a base category.
	=1 for one time switched, or 0 otherwise,
	=2 more than one time or 0 otherwise
Employment Sector	=1 Public Sector, 0 otherwise
Nature of Work	=1 faculty member, 0 for non-teaching staff
Area of Study	Engineering-Computer Sciences is the Base category.
	=1 Natural Sciences, 0 otherwise
	=1 Social and Management Sciences, 0 otherwise (Engineering and Computer Sciences, Social and Management Sciences) are merged in one category, respectively.
Marital Status	=1 Married, 0 otherwise
Funded Degree	=1 Funded, 0 otherwise
Gender	=1 Male, 0 otherwise

## Dependent variable

The monthly wage of an individual is considered a dependent variable because the research objective is to see the impact of levels of education on private lifetime economic returns. So, the monthly wage of faculty members is a dependent variable affected by the different levels of education and other exploratory variables.

## Independent variables

One of the independent primary explanatory variables is the level of education, which differentiates between Ph.D. and non-Ph.D. faculty. The second independent variable is the number of working years, like working experience. Therefore, it is essential to see the impact of job switching on economic returns, such as whether private returns are enhanced positively or not due to switching jobs from one institute to another. Meanwhile, private and public sector universities are included in the analysis, and *β*_5_ looks for the difference in returns.

## Simulation analysis for expected lifetime earnings

The simulation process calculates the expected undiscounted lifetime earnings of Ph.D. and non-Ph.D. faculty members. The simulation analysis is based on the survey data conducted in the different cities of Pakistan from four provinces, including Islamabad. The wages for different levels of education are estimated through regression analysis using the earnings function, and the coefficient value of education level is treated as wages with J years of schooling. Table 6 represents the description of the parameters used in the simulation process.

The following criteria are used to estimate the wages for levels of education,

- Ph.D. and non-Ph.D. faculty members in Pakistani Universities are selected.
- The average wages of the faculty members after a terminal degree are considered a starting wage.

Table 7 represents the simulation analysis results, which show Ph.D. and non-Ph.D. lifetime earnings of faculty for different years of education acquired by the faculty members. The starting working age is 25 years of non-Ph.D. and 29 years for Ph.D. faculty members. So, Fig. 8 gives the comparison of the Cumulative Net Present Value (CNPV) of lifetime earrings for a different level of education that shows the cumulative net earnings of non-Ph.D. is higher in the first seven years compared to holding a Ph.D. degree from Pakistani Universities and 13 years for foreign Ph.D. degree holders. Meanwhile, the annual expected undiscounted earnings are calculated for 32 & 36 years of working experience for Ph.D. and non-Ph.D. faculty members, respectively. The undiscounted lifetime earnings are equivalent to the annual undiscounted expected earnings of 32 and 36 years.

## Net Present Value (NPV) method

The Net Present Value (NPV) is the difference between the present value of cash inflows and cash outflows over time discounted by some rate. Therefore, the method incorporates the cost of acquiring education and lifetime earnings. As discussed earlier, the study calculates the NPV of a Ph.D. and non-Ph.D. lifetime earnings for faculty members in Pakistan. If NPV is positive (NPV > 0), the return on educational investment is positive. If NPV < 0, the return on education investment is negative. Finally, there will be no loss or profit if NPV = 0. The NPV is calculated using Eq. (7).

7 $$NPV = \mathop {\sum}\limits_t^T {E/\left( {1 + r} \right)^{ \wedge t} - Cost = 0}$$

Where E refers to earnings (annual returns in terms of wages) from job starting time to retirement age, r is the discount rate, t is the number of working years, and the cost is the total discounted cost of education plus foregone earnings. In addition, the cost of the Ph.D. degree is also adjusted for the inflation factor, using an average inflation rate of 6.07 in the last two decades (Ministry of Finance, Pakistan, 2020–21).

As discussed earlier, the objective is to calculate the net present value of lifetime earnings of Ph.D. over non-Ph.D. faculty members. So, Eq. (7) defines the whole process of calculating the Net Present Value (NPV). The right-hand side of Eq. (7) has two things: the present value of discounted lifetime earnings and the total cost. So, the first step will calculate the present value of lifetime earnings by using this part $\mathop {\sum}\nolimits_t^T {E/\left( {1 + r} \right)^{ \wedge t}}$, and the total cost will calculate in the second step. Finally, the total cost will be deducted from the discounted lifetime earnings to get the NPV. The NPV is calculated for both Ph.D. and non-Ph.D. faculty members, showing that the NPV of lifetime earnings is higher for Ph.D. faculty than non-Ph.D.

The undiscounted expected lifetime earnings of a Ph.D. faculty member are 67.8 and 70.4 million Pakistani rupees (PKR) that hold degrees from Pakistani and foreign universities, respectively. The Ph.D. degree cost is divided into two parts such as explicit (Direct Costs) and implicit costs. The explicit cost is PKR 6,73,327, and the implicit cost is PKR. 2.72 million. The total degree cost in Pakistan is almost PKR. 3.4 million, and most Ph.D. scholars earn, on average, PKR. 5,09,410 during the degree (Interim earnings). Therefore, after deducting interim earnings from the total cost, the total net cost of the degree in Pakistan is PKR. 2.89 million.

The total cost of a foreign Ph.D. degree is PKR. 7.51 million comprises explicit and implicit costs of PKR. 4.8 and 2.72 million, respectively. On the other hand, a foreign Ph.D. degree holder earns PKR. 1.12 million during the degree. Therefore, the total net cost is PKR. 6.38 million after deducting interim earnings from the total cost.

The human capital earning function is applied to calculate an individual’s lifetime private economic returns. Therefore, regression analysis (using earning function) and Net Present Value (NPV) methods are used. In step one, the earning function method is used for different education levels, experience, sectors of employment, and other control variables. In the second step, lifetime private economic returns of Ph.D. over non-Ph.D. faculty are calculated through the NPV method. In the first step, the forgone earnings were not in consideration, so in the second step, total cost (forgone earnings and direct expenditures) was subtracted from Present lifetime earnings.

Most studies have used the Internal rate of return (IRR) method, but the IRR method will not achieve the required results (As per study objectives) of this study, such as (Bashir and Iqbal, 2010). Many studies only used the earnings function for private returns to education, not for lifetime private economic returns (Haroon, 2015; Nasir, 1998; Javed and Arshad, 2013).

## Results and discussion

This section is divided into two parts; the first part presents descriptive statistics, while the second part presents the results of empirical analysis. Table 4 represents the relevant descriptive analysis, including average years of experience and the average time to complete the Ph.D. and non-Ph.D. degrees by gender.

**Table 4:** Descriptive analysis by gender.

Variables	Male		Female		Both	
	Mean	Std. Deviation	Mean	Std. Deviation	Mean	Std. Deviation
Experience	11.3	11.5	8.8	5.3	10.5	9.9
Avg. Ph.D. Degree Time (P)	4.6	1.04	4.9	1.27	4.7	1.13
Avg. Ph.D. Degree Time (F)	4	1	4.3	1.1	4	1.02
Avg. MPhil Degree Time (P)	2.2	0.39	2.2	0.41	2.2	0.4
Avg. MPhil Degree Time (F)	2	0.33	2.4	0.54	2.1	0.39

Source: Authors’ Estimations from survey data (P = Degree from Pakistan, F = Degree from Foreign).

Results showed that the maximum and minimum time to complete the Ph.D. degree is 8 and 3 years, respectively. On the other hand, the maximum completion time of an M.Phil. degree is 3.5 years. The degree completion time of Ph.D. and M.Phil. by gender is almost the same. It is identified that local Ph.D. degree holders take more time than foreign Ph.D. degree holders.

**Fig. 4:**
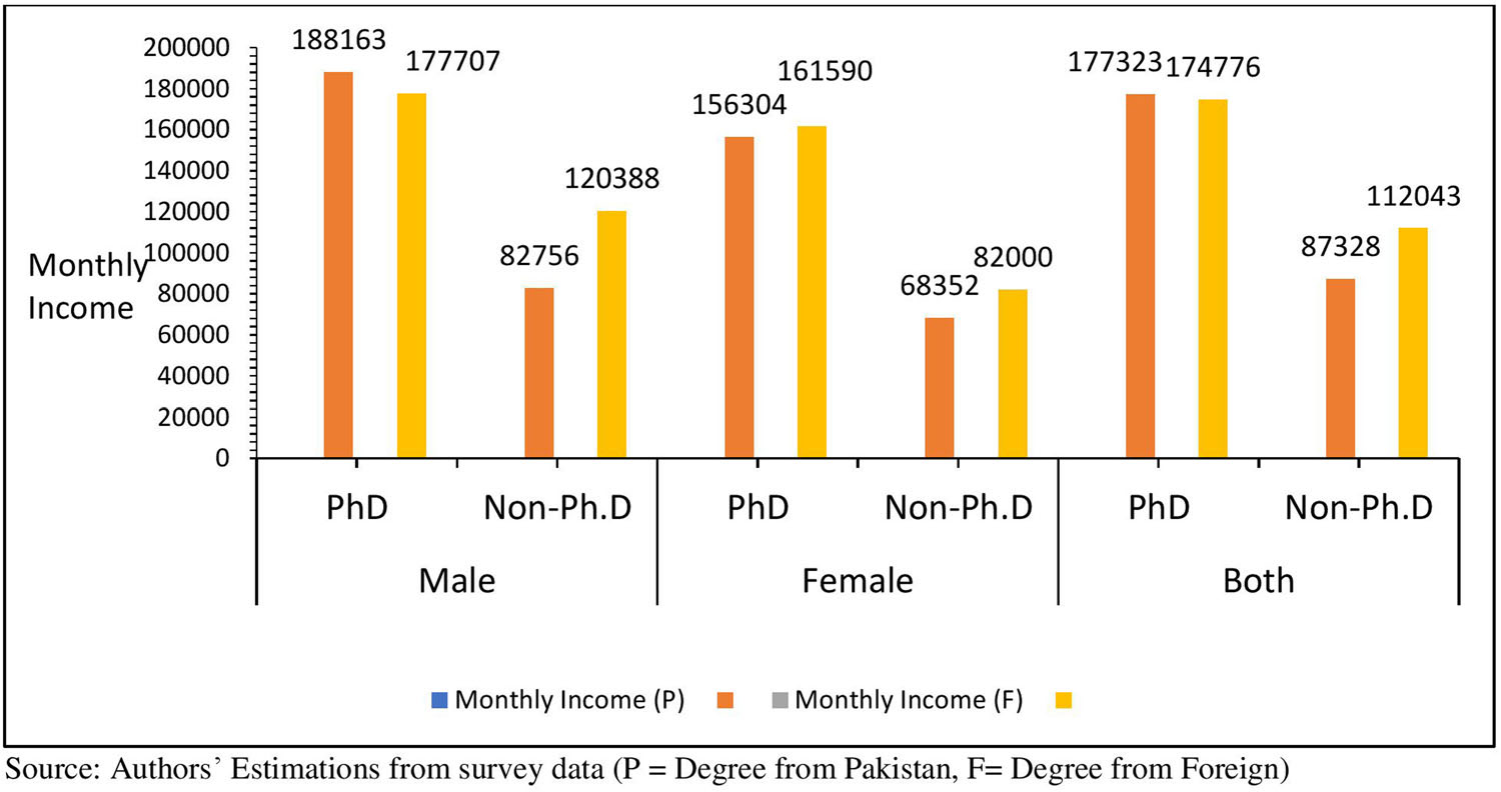
Average monthly income by education and gender (PKR). It shows the average monthly income of Ph.D. and non-Ph.D. male and female faculty members.

Figure 4 presents the average monthly income of Ph.D. and non-Ph.D. faculty members. The Ph.D. faculty members who have a degree from Pakistani universities earn almost double wages than non-Ph.D. faculty, whereas the average monthly income of foreign Ph.D. degree holders is nearly equivalent to that of Pakistani Ph.D. degree holders (See Fig. 4). However, the foreign non-Ph.D. degree holders are enjoying higher economic privileges than their domestic counterparts. Moreover, the difference in earnings between genders is a viable concern. It has been observed that male Ph.D. faculty members enjoy higher economic privileges than female Ph.D. faculty members.

**Fig. 5:**
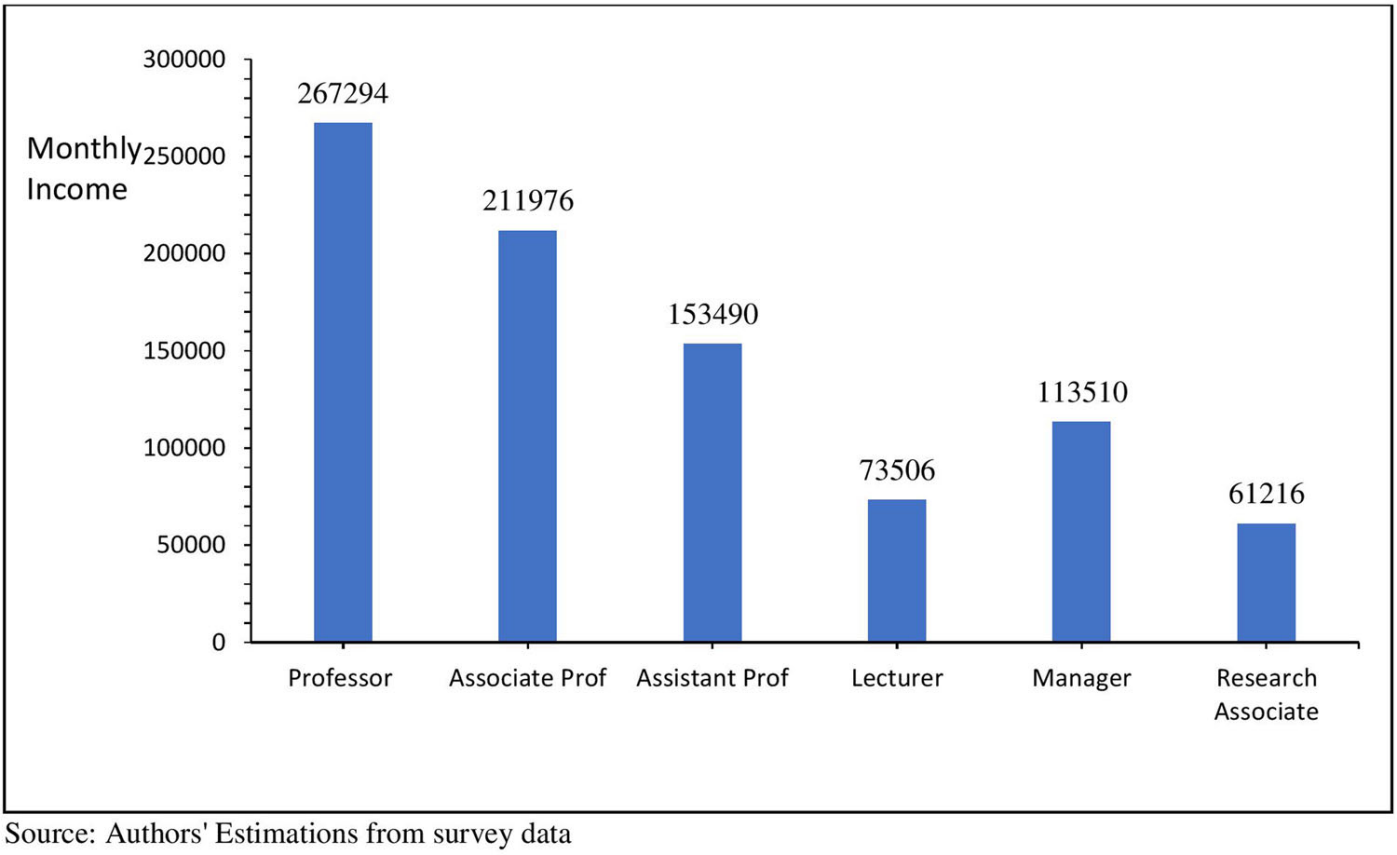
Average monthly income by designation (PKR). It shows the average monthly income of Professors, Associate Professors, Assistant Professors, and lecturers compared to managers and research associates.

Figure 5 represents the outcomes of average monthly income by the current designation of Ph.D. and non-Ph.D. faculty. The average monthly income of a professor is 26% higher than an associate professor, while the average income of an assistant professor is 38% less than that of an associate professor (See Fig. 5). The private economic returns of Ph.D. over non-Ph.D. faculty members are higher, indicating that a Ph.D. degree is significantly different in the private returns to education. The immediate designation in the academic field after the Ph.D. degree is assistant professor, so an assistant professor earns 108% higher than non-Ph.D. faculty.

Table 5 presents the average monthly income of Ph.D. and non-Ph.D. faculty across different sectors, including public and private sectors. It is worth noting here that, comparatively, returns in public sector universities are higher than that in the private sector. Regarding gender-wise returns on education, Table 5 shows that male Ph.D. faculty members in public and private sector universities have higher economic privileges than females. Male non-Ph.D. faculty members of public and private sector universities have 24% higher salaries than female faculty members.

**Table 5:** Average monthly income by private and public sector employment (Rs).

Faculty	Male		Female		Combined	
	Private Sector	Public Sector	Private Sector	Public Sector	Private Sector	Public Sector
Ph.D. Faculty	1,67,522	1,89,984	1,54,821	1,58,381	1,63,778	1,80,988
Non-Ph.D. Faculty	82,549	87,067	66,545	70,391	76,261	81,027

Source: Author’s Estimations from survey data.

**Fig. 6:**
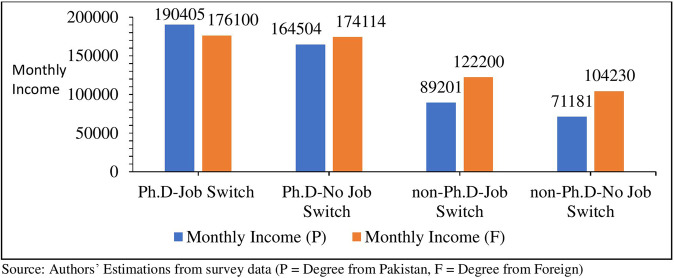
Average monthly income by the levels of education and job switching (PKR.). It compares the average monthly income of Ph.D. and non-Ph.D. faculty in case of job-switching and bifurcating them by faculty having Ph.D. from domestic and foreign universities.

Figure 6 shows the difference in the earnings of Ph.D. and non-Ph.D. faculty members who switched jobs during their professional careers. The Ph.D. faculty members who completed their degree from Pakistani universities and switched jobs from one institution to another earn 15.74% more wages than those who did not switch jobs (See Fig. 6). Meanwhile, the earnings of foreign Ph.D. degree holders are almost the same; only 1.14% earn more than those who do not switch jobs. As far as the earnings of non-Ph.D. faculty are concerned; they enjoy 25% and 17% higher wages than non-job-switchers.

**Fig. 7:**
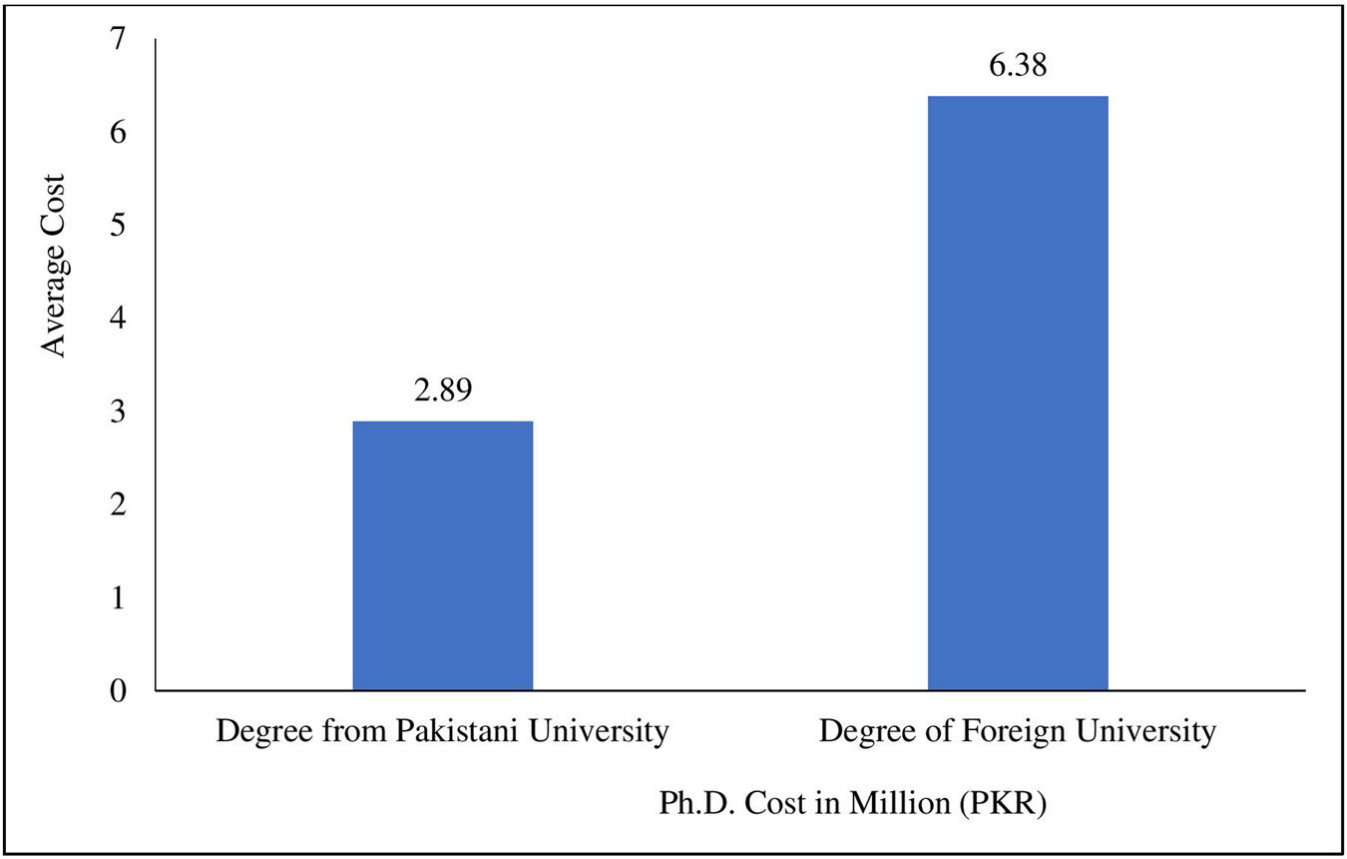
Country-wise average cost of Ph.D. degree. It shows the explicit and implicit costs (combined) of doing a Ph.D. from Pakistani universities compared to foreign universities. The cost of obtaining a Ph.D. from a foreign university is more than double that of a domestic university.

Figure 7 depicts the difference in the average cost of a Ph.D. degree in Pakistan and a foreign country. The explicit and implicit costs of completing a Ph.D. in Pakistan are 3.4 million, while a foreign degree costs 7.51 million. Moreover, the total average earnings during the Ph.D. (Interim earnings) degree from Pakistan are 0.5 million, while foreign country degree holders earn 1.1 million (See Fig. 7). Therefore, after the deduction of earnings (Interim earnings) from the total cost, the cost of a Ph.D. degree has decreased from 3.4 to 2.89 million for degree holders from Pakistani universities. Meanwhile, the total costs have decreased to 6.38 million for foreign-country degree holders.

### Empirical analysis

Equation (4) contains the following variables for the empirical analysis: level of education, experience, nature of work or job, employment sector, gender, etc. The modified version of earning function is used without using the log to obtain the absolute coefficient values because the study identifies the difference in lifetime earnings for different levels of education rather than one year increase in education. The Ordinary Least Square (OLS) method is applied for estimating private economic returns using earning function.

Table 6 represents regression results showing the impact of different explanatory variables on the private earnings of individuals. For example, education and experience positively affect private earnings, and the study’s results indicate that a Ph.D. degree holder earns higher monthly wages than a non-Ph.D. degree holder and results are also significant (Weiss and Landau, 1985). Therefore, the current results align with the previous studies’ outcomes.

**Table 6:** Level of education, experience, nature of work, and earnings.

Variables	Dependent Variable (Y)	
	Coef.	T & *P*-Value
Education Level (Ph.D. = 1)	70,326	17.49 (0.00)
Experience	2876	3.46 (0.00)
Sq. of Exp	−6.3	−0.25 (.8)
Sector of Emp	1604	0.44 (0.6)
Nature of Work	85	0.02 (0.9)
Funded degree	1041	0.22 (0.8)
Social & Mang. Sciences	−10324	−2.2 (0.02)
Natural Sciences	−10060	−2.11 (0.03)
Married	16,364	3.62 (0.00)
Gender (Male=1)	9042	2.48 (0.01)
Job Switching one time	−10617	−2.16 (0.03)
More than One Time	9738	2.33 (0.02)
Country of Degree	−6773	−1.23 (0.20)
Constant	52721	R2 = 0.54

Source: Authors’ Estimation from the survey data [Coefficient values are in PKR].

The monthly private earnings of Ph.D. over non-Ph.D. faculty are PKR. 70,326 higher, and the coefficient value of the level of education is significant. An extra year of experience increases monthly earnings by PKR. 2876 at a diminishing rate. The faculty members of the public sector earn higher wages than those working in the private sector universities, but the sector of employment variable shows insignificant results. Moreover, this study considers four major disciplines: social & management sciences, engineering, computer sciences, and natural sciences, with engineering & computer science disciplines as the base category.

The faculty members of the base category have higher earnings than the social & management and natural sciences employees with significant results. Along with significant results, married faculty members earn PKR. 16,364 more wages than unmarried faculty members (Ashraf, 2011). The Bruch pagan test is applied to detect the problem of heterogeneity that shows the P-value is less than .05, so the robust standard error has been applied as a remedial measure.

Meanwhile, the variance inflation factor (VIF) has been used to detect the multiclonality problem. The value of VIF is 2.79, which is less than 10, so the multicollinearity problem is not established. Furthermore, the Wu-Hausman test is used to detect the problem of endogeneity, which showed the *P*-value is not less than 0.05, so the endogeneity issue is not detected.

### Net present value of expected lifetime earnings

For calculating NPV, an average 7% interest rate on Pakistan Investment Bonds (PIBs) is used. The data on interest rates is accessible on the Census and Economic Information Center (CEIC) and Economic Survey of Pakistan 2021 (Ministry of Finance, 2020).

The first two rows of Table 7 give simulated values of undiscounted lifetime earnings and average monthly income, while rows three and four give PVs of earnings and the cost of doing a Ph.D. Then row five gives the NPV of the Ph.D. degree. The expected undiscounted lifetime earning of a local Ph.D. faculty is PKR. 67.8 million compared to PKR. 45.7 million for non-Ph.D. faculty.

**Table 7:** Earning’s net present value of Ph.D. over non-Ph.D. faculty by country (PKR).

Earnings	Non-PhD	Degree from Pakistan		Degree in foreign country	
		Ph.D.	Diff	Ph.D.	Diff
Undiscounted Lifetime Earnings	45.7 M	67.8 M	22.1 M	70.4 M	24.7 M
PV of Lifetime earnings @ r = 7% (PVLE)	14.7 M	24.46 M	9.74 M	25.5 M	10.77 M
PV of Cost (PVC)	0	2.89 M	- 2.89 M	6.38 M	- 6.38 M
NPV of Ph.D. Earning (NPV_phd_), (PVLE-PVC)	6.85 M			4.37 M	
Percentage change in Earnings of Ph.D. over non-Ph.D. faculty (NPV_phd_/PVLE_non-PhD_ *100)	46.5%			29.8%	
The net rate of return of Ph.D. Degree over the cost of a Ph.D.	237%			68.5%	

Source: Author’s Calculations from Survey data (Retirement Age is 60 Years).

The present value of lifetime earnings is Rs 9.74 million higher for a Ph.D. than for a non-Ph.D. faculty. After adjusting the present value of the cost of doing a Ph.D. Degree in Pakistan (Rs 2.89 million), the NPV of a Ph.D. degree is Rs 6.85 million, which is 46.5% higher than the earnings of the non-PhD faculty.

Compared to this, the foreign Ph.D. degree holder earns 29.8% higher returns than a non-Ph.D. faculty. This means that the NPV of a Ph.D. degree is higher for those who have completed Ph.D. degrees from domestic universities. This is due to the significantly higher cost of financing a Ph.D. degree from abroad. The results of Bashir and Iqbal (2010) study also indicate that the cost of a Ph.D. contributes to the reduction in private economic returns.

Table 8 shows the results of the net present value (NPV) of earnings of male Ph.D. over non-Ph.D. faculty. The net earnings of domestic and foreign Ph.D. degree holders are 49.7 and 32.9% higher than non-Ph.D. faculty respectively.

**Table 8:** Earning’s net present value of male Ph.D. over non-Ph.D. faculty (PKR).

Earnings	Non-Ph.D.	Degree from Pakistan		Degree in foreign country	
		Ph.D.	Diff	Ph.D.	Diff
Undiscounted Lifetime Earnings	45.7 M	69 M	23.3 M	71 M	25.3 M
PV of Lifetime earnings @ r = 7% (PVLE)	14.7 M	24.9 M	10.2 M	25.9 M	11.2 M
PV of Cost (PVC)	0	2.89 M	2.89 M	6.3 M	6.3 M
NPV of Ph.D. Earning (NPV_phd_), (PVLE-PVC)	7.3 M			4.8 M	
Percentage change in Earnings of Ph.D. over non-Ph.D. faculty (NPV_phd_/PVLE_non-PhD_ *100)	49.70%			32.90%	
The net rate of return of Ph.D. Degree over the cost of a Ph.D.	253.6%			76%	

Source: Authors’ Calculations from Survey data (Retirement Age is 60 Years).

The earnings of male Ph.D. faculty are 3% higher than the combined results (Table 7) of both males and females. Javed and Arshad (2013) calculated that male teachers earn more than female faculty members in the education sector of Pakistan.

Table 9 represents the NPV of earnings of female Ph.D. over non-Ph.D. faculty by country of degree. The net earnings of female Ph.D. faculty are 40.4% higher than non-Ph.D. faculty who hold a degree from Pakistani universities, and 23.6% higher earnings of those female Ph.D. faculty members who earned their Ph.D. degrees from abroad. Moreover, the male faculty earns higher lifetime economic returns than female faculty members. The private economic returns of male Ph.D. faculty who completed their degree from Pakistani universities earn 9.3% higher than female faculty members. Khan et al. (2012) examined the gender disparity in the economic returns of higher education. The study’s findings revealed that at a higher level of education (Postgraduate), the male private economic returns are higher than female returns. The research of Shaukat et al. (2014) also supports the current study’s results that gender disparity exists in universities.

**Table 9:** Earning’s net present value of female Ph.D. over non-Ph.D. faculty.

Earnings	Non-Ph.D.	Degree From Pakistan		Degree in Foreign Country	
		Ph.D.	Diff	Ph.D.	Diff
Undiscounted Lifetime Earnings	45.7 M	65.5 M	19.8 M	68.1 M	22.4 M
PV of Lifetime earnings @ r = 7% (PVLE)	14.7 M	23.5	8.8 M	24.6 M	9.8 M
PV of Cost (PVC)	0	2.89 M	2.89 M	6.3 M	6.3 M
NPV of Ph.D. Earning (NPV_phd_), (PVLE-PVC)	5.9 M			3.4 M	
Percentage change in Earnings of Ph.D. over non-Ph.D. faculty (NPV_phd_/PVLE_non-PhD_ *100)	40.40%			23.60%	
The net rate of return of Ph.D. Degree over the cost of a Ph.D.	206.1%			55.5%	

Source: Author’s Calculations from Survey data (Retirement Age is 60 Years).

### Cumulative net present value of expected lifetime earnings

**Fig. 8:**
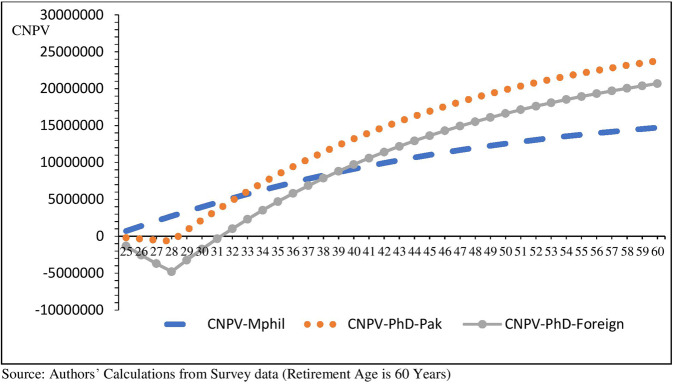
Cumulative Net Present Value (CNPV) of expected lifetime earnings by degree. It compares the CNPV of domestic, foreign Ph.D., and non-Ph.D. faculty members.

Figure 8 compares earnings in terms of cumulative NPV of expected lifetime income of Ph.D. over non-Ph.D. faculty. The starting age of joining the labor market is 25 years for non-Ph.D. faculty and 29 for the Ph.D. faculty. The cumulative net present value line of a Ph.D. is decreasing due to the cost of education.

The cumulative net present value of lifetime earnings of non-Ph.D. faculty is higher than Ph.D. till the age of 32 and 38 years depending upon the source of the Ph.D. degree (See Fig. 8). Domestic Ph.D. degree holders have a higher cumulative NPV of earnings than their foreign counterparts. This is mainly due to the higher cost of a foreign Ph.D. degree.

### Sensitivity analysis

Sensitivity analysis is based on relaxing assumptions and restrictions to see their impact on study outcomes. We evaluate the impact of the following variables on the NPV of lifetime earnings:

1. **Changes in retirement age:** The analysis of private returns of Ph.D. over non-Ph.D. faculty is carried out from 55 to 65 years of retirement age.
2. **Changes in Year of Completion of Ph.D. Degree:** Here, we find the effect on NPV of earnings for Ph.D. faculty when it takes extra years to complete the degree.
3. **Changes in discount rate:** The effect of changing the discount rate from 7% to 3 and 11%.

Table 10 represents the results of NPV of earnings of a Ph.D. degree at alternate retirement age. Again, this will not affect the costs of a Ph.D. degree but only the NPV of expected earnings.

**Table 10:** NPV of earnings of Ph.D. degree at alternative retirement ages.

Retirement Ages	Earnings	Ph.D. degree from Pakistan	Ph.D. degree from Foreign
55 Years	NPV of Ph.D. earning (NPV_phd_)	6.1 M	3.58 M
	Percentage change in earnings of Ph.D. over non-Ph.D. faculty	44.30%	26%
	The net rate of return of a Ph.D. degree over the cost of a Ph.D.	111%	−43%
	Percentage change in earnings relative to 60 Years	−2.20%	−3.80%
60 Years	NPV of Ph.D. earning (NPV_phd_)	6.85 M	4.37 M
	Percentage change in earnings of Ph.D. over non-Ph.D. faculty	46.50%	29.80%
	The net rate of return of a Ph.D. degree over the cost of a Ph.D.	237%	68.50%
65 Years	NPV of Ph.D. earning (NPV_phd_)	7.4 M	5.1 M
	Percentage change in earnings Ph.D. over non-Ph.D. faculty	47.90%	33%
	The net rate of return Ph.D. degree over the cost of a Ph.D.	256.20%	79.90%
	Percentage change in earnings relative to 60 Years	1.40%	3.20%

Source: Authors’ Calculations from Survey data (Retirement Age is 55 Years).

If a Ph.D. degree is completed in Pakistan, the lifetime earnings have decreased from 67.8 million to 54.9 for Ph.D. faculty in the context where the retirement age has been alternated from 60 to 55 years. As a result, the NPV of lifetime earnings of a Ph.D. degree has decreased from PKR. 6.85 to 6.1 million, while the costs are not changed because the changes in the retirement age have only affected the earnings. The net earnings of Ph.D. over non-Ph.D. faculty have decreased by 2.2 and 3.8% when the Ph.D. degree was completed in Pakistan and foreign countries.

Secondly, we investigated the percentage change in NPV earnings for Ph.D. over non-Ph.D. faculty with a retirement age of 65 years instead of 60 years. We found that the net lifetime earnings increased by 1.4 and 3.2% for Ph.D. faculty who completed their degree in Pakistan and Foreign countries, respectively, if the retirement age is increased to 65. Meanwhile, the net earnings of Ph.D. faculty are PKR. 7.4 million for having degrees from Pakistan: however, the earnings of foreign Ph.D. faculty are PKR. 5.1 million.

Table 11 presents the impact of percentage change in NPV of earnings when a Ph.D. candidate takes extra years to complete the Ph.D. degree. In fact, as per the rules Ph.D. degree has a minimum time frame to complete it, but the net earnings could be changed due to a marginal change in the degree completion time. Meanwhile, there is a tradeoff between the years of education and the working life span, so one extra year to complete the degree will magnify the implicit and explicit costs. Consequently, our study shows the lifetime net earnings of Ph.D. over non-Ph.D. faculty decreases by 5.66 and 5.83% for Pakistani and foreign countries degree holders, respectively. The study by Bashir and Iqbal (2010) also endorsed these results, such as one or two extra years will negatively impact the lifetime private economic returns of a Ph.D. degree holder.

**Table 11:** Effect of delay in completion of the Ph.D. degree on NPV of earnings.

Delays in Years	Earnings	Ph.D. degree from Pakistan	Ph.D. degree from Foreign
1	NPV of Ph.D. earning (NPV_phd_)	6.01M	3.52M
	Percentage change in earnings of Ph.D. over non-Ph.D. faculty	40.84%	23.97%
	The net rate of return of a Ph.D. degree over the cost of a Ph.D.	175%	50.89%
	Effect of dallying Ph.D. degree on the lifetime net earnings	−5.66%	−5.83%
2	NPV of Ph.D. earning (NPV_phd_)	5.156M	2.66M
	Percentage change in earnings of Ph.D. over non-Ph.D. faculty	35.04%	18.10%
	The net rate of return of a Ph.D. degree over the cost of a Ph.D.	130.40%	35.71%
	Effect of dallying Ph.D. degree on the lifetime net earnings	−11.46%	−11.70%

Source: Author’s Calculations from survey data.

In the same way, Table 12 above presents the difference in results of net lifetime earnings of Ph.D. faculty at 3 and 11% of interest rates. Again, the changes in outcomes are compared with the already estimated results with a 7% interest rate.

**Table 12:** NPV of earnings of Ph.D. over non-PhD faculty at different interest rates.

Interest rates	Earnings	Ph.D. degree from Pakistan	Ph.D. degree from Foreign
3%	NPV of Ph.D. earning (NPV_phd_)	10.9 M	8.7 M
	Percentage change in earnings of Ph.D. over non-Ph.D. faculty	40.10%	32%
	The net rate of return of a Ph.D. degree over the cost of a Ph.D.	345.7%	125%
	Effect of different interest rates on the lifetime net earnings	−6.40%	2.20%
7%	NPV of Ph.D. earning (NPV_phd_)	6.85 M	4.37 M
	Percentage change in earnings of Ph.D. over non-Ph.D. faculty	46.50%	29.80%
	The net rate of return of a Ph.D. degree over the cost of a Ph.D.	237.00%	68.50%
11%	NPV of Ph.D. earning (NPV_phd_)	4.42 M	1.92 M
	Percentage change in earnings of Ph.D. over non-Ph.D. faculty	47.90%	20.88%
	The net rate of return of a Ph.D. degree over the cost of a Ph.D.	167.10%	32.10%
	Effect of different interest rates on the lifetime net earnings	1.40%	-8.92%

Source: Author’s Calculations from survey data.

## Conclusion

The result of the study is an insight that the private rate of returns of Ph.D. faculty is higher than non-Ph.D. faculty, although the non-Ph.D. faculty have a larger working life span. Finally, Ph.D. degree holders from Pakistani universities have higher lifetime economic returns than foreign-country degree holders because the costs of the foreign Ph.D. degree are much higher than those of domestic. Meanwhile, the lifetime economic returns are reduced if a scholar takes one extra year to complete the Ph.D. degree. On the other hand, the lifetime returns will increase by 1.4 and 3.2% due to an increase in the retirement age from 60 to 65 for domestic and foreign Ph.D. degree holders.

Policy implications of the current study are that the lifetime private economic returns should be improved for Ph.D. degree holders in the following ways by the policymakers.

1. The pay scale of the Ph.D. degree holders should improve specifically for the foreign degree holders because they face higher costs.
2. The interim earnings of a Ph.D. degree should be higher than now, so the government should plan fruitful policies for Ph.D. scholars.
3. The retirement age of Ph.D. degree holders should increase from 60 to 65 years.

Apart from educational policymakers, the study’s findings will also help Ph.D. applicants looking for admission to postgraduate programs. Analysis of the current study centered on cross-sectional data such as faculty members who completed their degrees between 2000 to 2021. Moreover, due to covid-19 situation, the study is based on the academic sector, but in the future, any researcher can make a comparison of private economic returns between the academic and research and development (R&D) sectors.

## Data Availability

The authors have no permission to share the data because it has some confidential information; however, it is available on request from the corresponding author.
